# The Role of 3D Virtual Anatomy and Scanning Environmental Electron Microscopy in Understanding Morphology and Pathology of Ancient Bodies

**DOI:** 10.3390/tomography11010005

**Published:** 2025-01-03

**Authors:** Sara Salucci, Mirko Traversari, Laura Valentini, Ilaria Versari, Luca Ventura, Emanuela Giampalma, Elena Righi, Enrico Petrella, Pietro Gobbi, Gianandrea Pasquinelli, Irene Faenza

**Affiliations:** 1 Department of Biomedical and NeuroMotor Sciences (DIBINEM), University of Bologna, 40126 Bologna, Italy; ilaria.versari4@unibo.it (I.V.); irene.faenza2@unibo.it (I.F.); 2 Department of Medical and Surgical Sciences (DIMEC), University of Bologna, 40126 Bologna, Italy; gianandr.pasquinelli@unibo.it; 3 Department of Biomolecular Sciences (DiSB), Urbino University Carlo Bo, 61029 Urbino, Italy; laura.valentini@uniurb.it (L.V.); pietro.gobbi@uniurb.it (P.G.); 4 Division of Pathology, San Salvatore Hospital, 67100 L’Aquila, Italy; lucaventura67@gmail.com; 5 Department of Biotechnological and Applied Clinical Sciences, University of L’Aquila, 67100 L’Aquila, Italy; 6 Department of Radiology, AUSL Romagna, Morgagni-Pierantoni City Hospital, 47100 Forli, Italy; emanuela.giampalma@auslromagna.it (E.G.); elena.righi@comune.modena.it (E.R.); epetrella@sirm.org (E.P.)

**Keywords:** human mummies, virtual anatomical table, environmental scanning electron microscopy, mummy body images, virtopsy

## Abstract

Background/Objectives: Mummy studies allow to reconstruct the characteristic of a population in a specific spatiotemporal context, in terms of living conditions, pathologies and death. Radiology represents an efficient diagnostic technique able to establish the preservation state of mummified organs and to estimate the patient's pathological conditions. However, the radiological approach shows some limitations. Although bone structures are easy to differentiate, soft tissue components are much more challenging, especially when they overlap. For this reason, computed tomography, a well-established approach that achieves optimal image contrast and three-dimensional reconstruction, has been introduced. This original article focuses attention on the role of virtual dissection as a promising technology for exploring human mummy anatomy and considers the potential of environmental scanning electron microscopy and X-ray spectroscopy as complementary approaches useful to understand the state of preservation of mummified remains. Methods: Ancient mummy corps have been analyzed through Anatomage Table 10 and environmental scanning electron microscope equipped with X-ray spectrometer; Results: Anatomage Table 10 through various volumetric renderings allows us to describe spine alteration due to osteoarthritis, dental state, and other clinical-pathological characteristics of different mummies. Environmental scanning electron microscope, with the advantage of observing mummified samples without prior specimen preparation, details on the state of tissue fragments. Skin, tendon and muscle show a preserved morphology and keratinocytes, collagen fibers and tendon structures are easily recognizable. Furthermore, X-ray spectrometer reveals in our tissue remains, the presence of compounds related to soil contamination. This investigation identifies a plethora of organic and inorganic substances where the mummies were found, providing crucial information about the mummification environment. Conclusions: These morphological and analytical techniques make it possible to study mummified bodies and describe their anatomical details in real size, in a non-invasive and innovative way, demonstrating that these interdisciplinary approaches could have great potential for improving knowledge in the study of ancient corpses.

## 1. Introduction

Arthur Aufderheide, a well-known pathologist and a famous pioneer in the study of ancient-world diseases, presented this definition for mummies: “In adjectival form the term mummified human can be applied conveniently to even the smallest non-skeletal fragment of a human body surviving a post-mortem interval long enough normally to anticipate complete decay” [1]. The charm of mummies has always attracted the interest of researchers [2,3], and natural mummification, a preservative process leading to the desiccation, brittleness, and shrinkage of the skin and body tissues [4], may be an interesting source of investigation.

Paleopathology and bioarcheology are disciplines involved in the study of mummies. However, the analysis of the human body cannot exclude anatomical observation. Since the 16th century, the development of pathological anatomy can be seen as a necessary step toward understanding the world of mummies [5]. To date, mummy research involves multidisciplinary approaches and, more recently, three-dimensional imaging resources. Computed tomography (CT), introduced in the 1970s, is an alternative technique to conventional mummy dissection, offering some advantages such as high spatial resolution, image contrast, a three-dimensional reconstruction, and others [6,7,8,9]. CT scan images allow paleopathologists to know the characteristics of an ancient population, including their diseases, the causes of death, bone alterations, and dental conditions [10,11]. Furthermore, this tomographic approach allows a more accurate description of anatomical structures than conventional X-ray analysis without artificial alterations caused by embalming material. CT can therefore be described as a non-destructive dissection technique, and CT images can be a starting point for innovative approaches, such as virtual reality, in the study of the human body.

Among virtual imaging tools, Anatomage Table represents an alternative way of describing human anatomy. It is a virtual dissection table that is useful for the training of medical students [12] but with digital possibilities that extend its range of application. The anatomy and physiology of the human body can be studied thanks to Korean and Asian cadavers loaded in the Anatomage Table 10 software by simulating a virtual dissection in all anatomical projections [13]. The table is supplemented with preinstalled medical image volumes, including CT and MRI. It is possible to upload image volumes of real patients (MRI or CT scans) by modifying the file extension in Digital Imaging and Communications in Medicine (DICOM) format, including those of human mummy bodies. The Anatomage tool can display a real mummified cadaver from DICOM format and show real patient data on a real-size scale by using the same CT technology. It can perform accurate and digital 3D reconstruction of the human body, and it is possible to perform dissections on the virtual patient or cadaver for internal inspection and segmentation. This virtual anatomical approach has many advantages, including providing a safe working environment that is free of chemicals or hazardous materials that might harm researchers during mummy dissection [14].

CT images and virtual dissection enable us to study mummies from a macroscopic point of view, but microscopic observations reveal a certain degree of deterioration that may not be visible at the macroscopic level. Therefore, morphological analyses represent an important component to better describe and detail the degradation process of mummified tissues and to assess their condition. In the literature, the morphological observation of mummified tissues is usually found to be performed by conventional scanning electron microscopy (SEM). However, this technique shows some limitations concerning sample preparation [15]. Instead, environmental scanning electron microscopy (ESEM) represents a valid alternative to conventional SEM, and, by working at low-vacuum conditions, it offers the possibility to operate on non-conductive samples without any preparation or hydration of specimens. After ESEM observations, specimens can be processed for further analyses since this microscopical approach is nondestructive. ESEM captures impressive images of the micro- and the nano-world; thus, it can provide interesting information about the mummification world. However, the study of mummy remains requires several analytic approaches due to the fact that mummified tissues can be modified by anthropic or natural alterations, e.g., the presence of toxic elements coming from the environment or of some cosmetic treatments applied before or during the funerary ritual [16]. Investigations of archaeological remains through X-ray spectroscopy (EDX analysis), which can identify a plethora of organic and inorganic substances, appear to be a crucial aspect in understanding the mummification process. This work aims to demonstrate that multidisciplinary approaches, including virtual dissection, microscopical observations, and EDX analysis, are indispensable in the study of mummies. Furthermore, this manuscript illustrates for the first time the great potential of Anatomage Table in contributing to the knowledge of this fascinating topic.

## 2. Materials and Methods

### 2.1. Samples

This study starts from CT scan images (no static multi-energy or SME) of three mummies discovered in a church crypt in the village of Roccapelago (Modena, Italy) and dating back to the 16th–18th century. These bodies are visible at the “Museo delle Mummie di Roccapelago”, a public museum built inside the San Paul’s Conversion Church of Roccapelago in Pievepelago. CT scan images were acquired by the Department of Radiology of the GB Morgagni Hospital in Forlì (1.25 slice thickness, interval of reconstruction of 0.7 mm, 120 kV, and 140–300 mA).

Moreover, CT scan images (no SME) and the morphological analysis of tissue remains have been performed on a Peruvian mummy from the Necropolis of Ancón, currently preserved in the section on Ancient Peru in the Civic Museum of Modena. The mummy belongs to a valuable collection of materials recovered by Antonio Boccolari and Paolo Parenti, two officers of the Italian navy who were part of the crew of the Corvetta Vettor-Pisani, during their voyage to circumnavigate the globe toward the end of the 19th century. The CT scan was performed at the Policlinico of Modena Hospital (2.5 mm slice thickness, interval of reconstruction of 0.7 mm, 120 kV, and 250 mA). All CT images have been interpreted following the recommendation suggested by O’Brien et al. (2009) [17]. The biological profile (estimation of sex, age, and stature) was performed in a virtual environment, through the analysis of the shape of the pelvis and skull. In addition, evaluation has been performed of the developmental or degeneration traits of bones and teeth and the maturation of ossification centers, adhering to accredited international standards [18,19,20].

### 2.2. Anatomage Table

CT scan files were converted to DICOM format, loaded into Anatomage Table version 10.0, and displayed on its 84-inch touchscreen [12].

**Figure 1:**
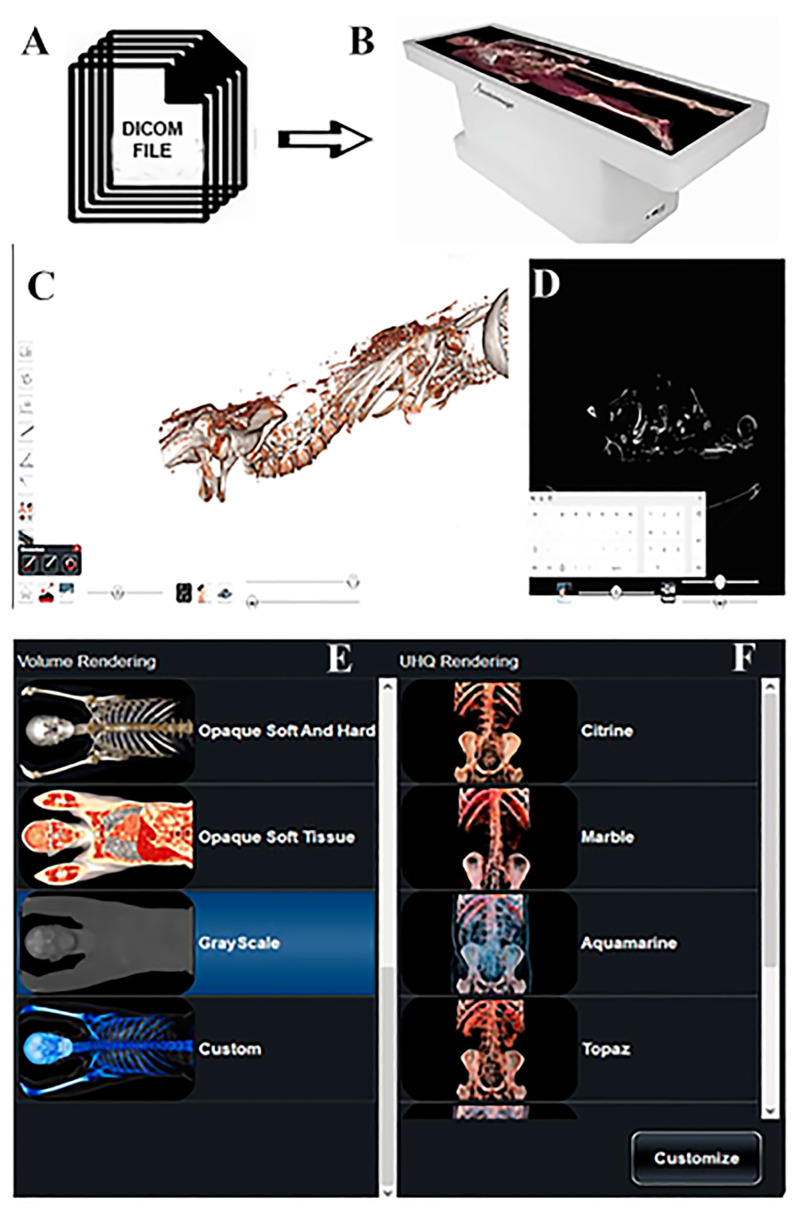
A schematic illustration of mummy body reconstruction. DICOM files (**A**) are loaded in Anatomage Table (**B**). The mummy (**C**) has been reconstructed by using opaque soft tissue volume rendering and it can be also visualized in CT scan modality (**D**). Some volume renderings are visible in (**E**,**F**).

As illustrated in Figure 1, DICOM files were opened through in vivo 3D imaging software (Anatomage Table 10). The use of specific volumetric rendering (filters) permitted us to appreciate both hard and soft tissues. Therefore, virtual dissection is not limited to bone structures, but can also analyze the remains of visceral structures, tendons, and ligaments; as well, details related to clothes can also be studied. Researchers, after applying the filter, manually moved the intensity of each volumetric rendering, thus revealing the anatomical and pathological characteristics of the picture gradually. Exploiting the computed tomography scan modality (CT), mummified bodies were observed on all anatomical projections. Therefore, Anatomage Table helped researchers to virtually dissect a life-sized human mummy through a digital hands-on approach.

### 2.3. ESEM and EDX

Mummified tissues were deposited on aluminum specimen stubs [21], which were previously covered with a conductive carbon adhesive disk [22] (TAAB Ltd., Calleva Park, Aldermaston, Berks, UK). An FEI Quanta 200 FEG Environmental Scanning Electron Microscope (FEI, Hillsboro, OR, USA), equipped with an energy-dispersive X-ray spectrometer (EDAX Inc., Mahwah, NJ, USA), was used to evaluate tissue morphology and its preservation, and to analyze chemical characteristics with the aim to identify specific elements that could have favored the mummification processes. The analyses were performed by using a focalized electron beam at a vacuum-electron-gun pressure of 5.0 × 10^−6^ mbar. The ESEM was used in low-vacuum mode with a specimen chamber pressure set at 0.80 mbar, an accelerating voltage of 15–20 kV, and a magnification ranging between 700 and 5000×. The images were obtained by means of a back-scattered electron detector. The spectrometer unit (EDX) was equipped with an ECON (Edax Carbon Oxygen Nitrogen) 6 utw X-ray detector and Genesis Analysis software (Genesis 1.8.1).

Each analysis was performed with a time count of 100 s, a spot size value between 3.6 and 4.0, and an amplification time of 51, while the probe current was 290 μA [23].

## 3. Results

Mummified bodies loaded in Anatomage Table 10 as DICOM files (Figure 1) have been described, starting from their clothing, and were subsequently “stripped” to display the anatomical structures.

**Figure 2:**
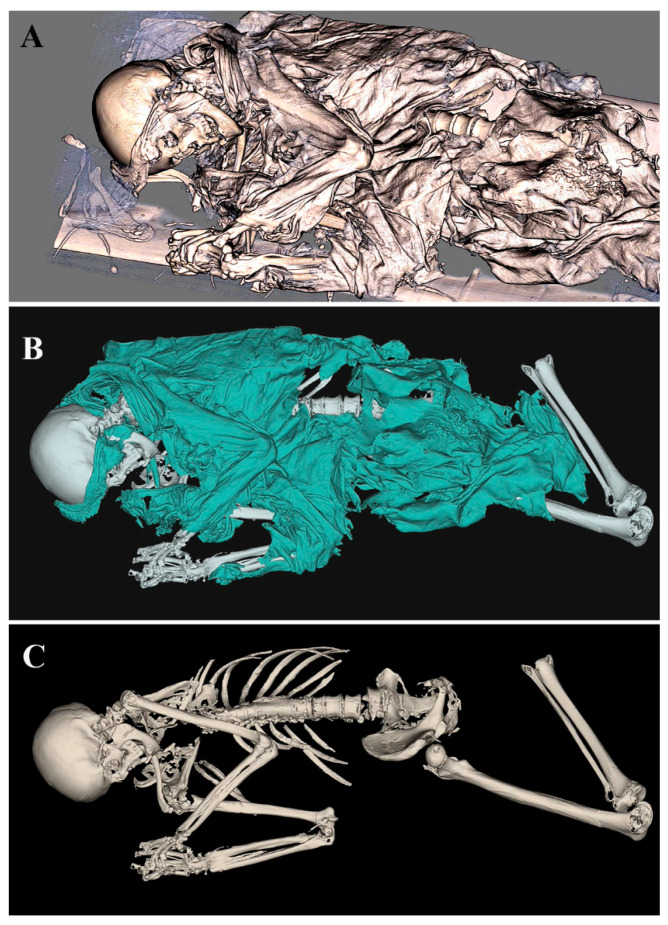
ID.54-23 mummy reconstructed using opaque soft tissue filter. (**A**) The mummy as it appeared on the support used for transport. (**B**) Textiles were highlighted. (**C**) The mummy virtually stripped.

Id.54-23 was positioned with joined hands and bent knees. The mummy had been reconstructed in 3D using the opaque soft tissue filter (Figure 2A), and she appeared lying on the table used to obtain radiological and tomographic images.

**Figure 3:**
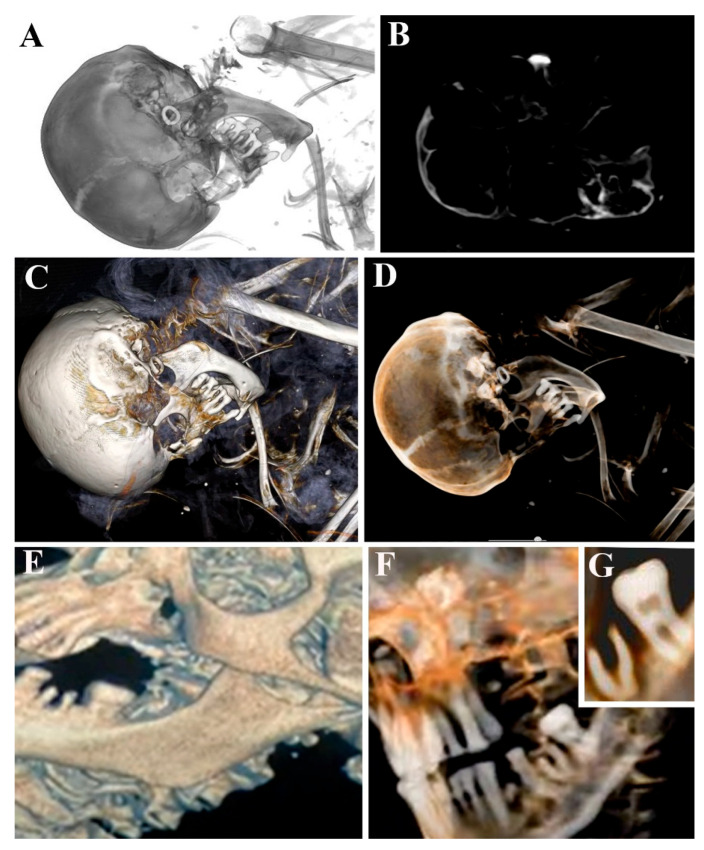
ID.54-23 mummy skull observed through gray-scale filter with the corresponding CT image and transparent, soft, and hard tissue rendering, which reveals the earring (**A**–**D**). Moreover, dental state and care presence can be described by means of opaque and transparent soft tissue filters (**E**–**G**).

Id.54-23 was partially wrapped in a conventional green dress and had a band that covered the forehead and ears (Figure 2B). Afterwards, the mummy was stripped and observed through deeper coronal sections (Figure 2C). As shown in Figure 3, skeletal components, such as skull, upper limb bones, and spinal column can be appreciated. The sex estimated through the observation of the pelvic and cranium morphology is female, and the estimated age at death, based on the observed indicators, is senile (dental attrition score > 50 years); through to the virtual autopsy, it was possible to observe the entire spinal column affected by severe marginal lipping, which, considering the age of the individual, could be attributable to osteoarthritis.

At this level (Figure 3A), due to the possibility of removing coronal planes and applying the gray-scale filter, a detail in the left ear appeared (Figure 3B). The radiological approaches and CT had not revealed this detail before since it was hidden by the bandage, as shown in the previous image. It was an earring, whose presence was also detected in the corresponding CT scan image (Figure 3B). The earring, in fact, showed a very different density compared to that of the surrounding bone, and it could be enhanced by other volumetric renderings, such as opaque and transparent soft and hard tissue filters (Figure 3C,D). Furthermore, these filters appear useful for highlighting the dental state (Figure 3E) and for studying the roots of the teeth and the presence of dental care (Figure 3F,G). The cavities and numerous alveolar resorptions in the mandibula reflected pathological changes, which might be due to poor or ineffective oral hygiene resulting from a diet high in unrefined foods containing simple sugars; moreover, teeth loss occurred long before death.

**Figure 4:**
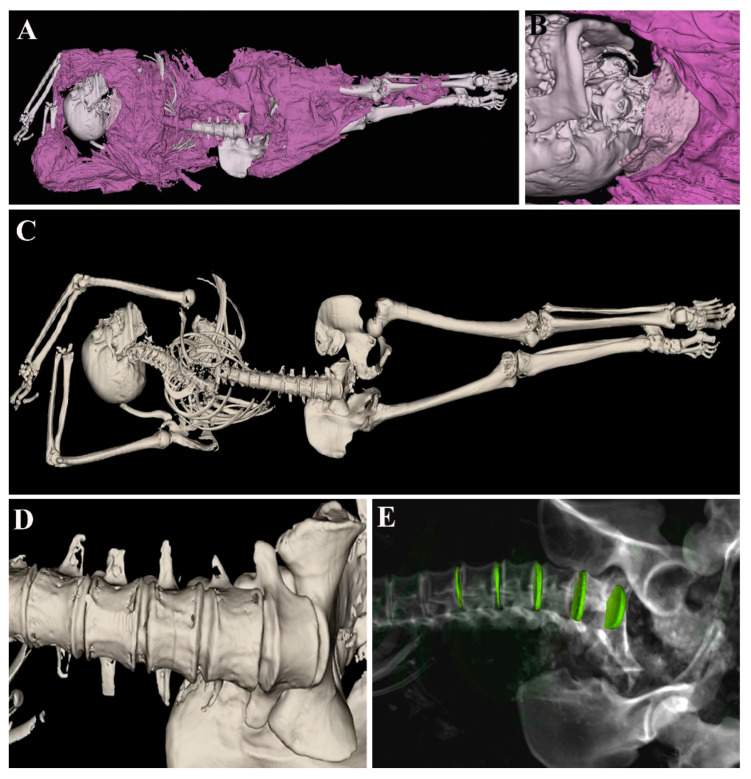
ID.50-23 mummy observed through opaque soft tissue filter (**A**). The filter allows one to appreciate some details like a lace collar (**B**). After removing the dress, the entire skeleton is visible (**C**) and shows some paramorphism of lumbar vertebrae (**D**), which can be better described by inserting gray-scale rendering (**E**).

The second mummy, ID.50-23, is known as “the dancer” since she was found in a fifth ballet position. The sex estimated through the observation of the pelvic and cranium morphology is female, and the estimated age at death based on the observed indicators is adult (dental attrition score 20–29 years). By applying the opaque soft tissue filter, she was found to wear a conventional fuchsia dress (Figure 4A) with lace in the collar (Figure 4B). This latter can be described through Anatomage Table from all points of view without physically damaging the mummy. The paramorphism of the skeletal apparatus (Figure 4B) can be observed by stripping the mummy. In particular, the left asymmetry of the lumbar vertebrae is visible in Figure 4C. By inserting a transparent gray-scale filter, fibrocartilaginous discs were colored and, thus, the asymmetry appeared more obvious (Figure 4D).

**Figure 5:**
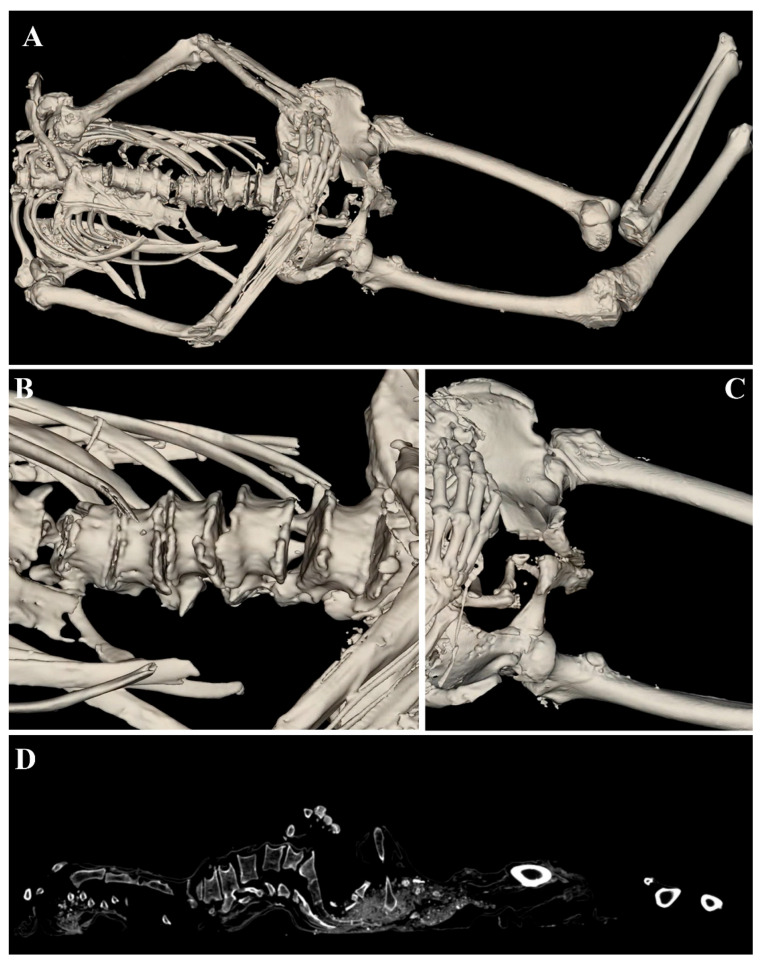
ID.59-23 mummy reconstructed using opaque soft tissue filter (**A**). Bone segment degeneration correlated to lumbar spine (**B**) and femoral bone (**C**) appear. In (**D**), we show the corresponding CT image.

The third mummified body, ID.59-23, was found without a skull. It is a mummy of a man, reconstructed at the anatomical table by applying the opaque soft tissue filter that allowed us to study the bone characteristics. The sex identified through the observation of the pelvic morphology is male, and the estimated age at death based on the observed indicators is senile (pubic symphysis score > 50 years) This Id presented signs of arthritis in the hip, knee, and both humeral heads. A significant bilateral enthesopathy of the gluteus medius muscle was also found at the insertion point with the iliac crest, likely due to functional overload. Furthermore, degenerative alterations of the lumbar spine (Figure 5B) and the sclerosis of the glena, combined with the irregularity of greater tuberosity (Figure 5C), can be observed. The spinal column was severely affected by degenerative processes, and, in fact, significant vertebral lipping was evident, especially on the lumbar segment, as well as numerous bridging osteophytes, which always affected this section. Spine degeneration was confirmed through the table using the CT scan modality (Figure 5D).

**Figure 6:**
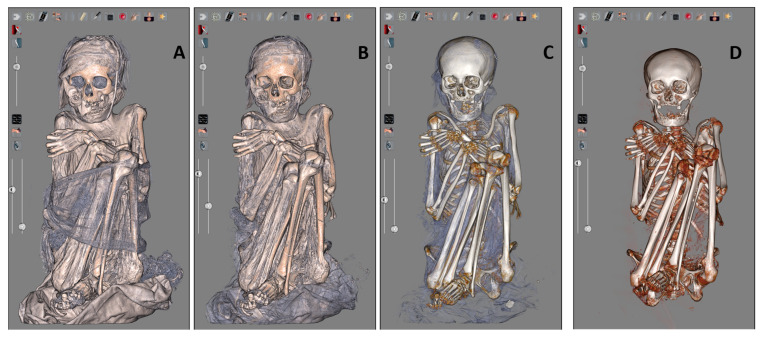
The Peruvian mummy during the virtual evaluation phases: with the textiles still visible (**A**); with the bandages removed (**B**); with the bone structure distinguishable (**C**); with only the skeleton visible (**D**), with possibility to evaluate the sex dimorphic characteristics and apply anthropometric analysis.

Finally, through the virtual dissection, we analyzed a Peruvian mummy from the 13th–14th century from the Necropolis of Ancón (Figure 6). We observed the mummy wrapped in her robe (Figure 6A,B). Moving from the superficial layers to the deeper ones, the mummy appeared completely naked, and we highlighted the components of the skeletal system (Figure 6C,D), defined the sex of the mummy, and measured the anthropometric parameters. The sex identified through the observation of the pelvic and cranium morphology is female, and the estimated age at death based on the observed indicators is young adult (fusion of ossification centers score 17–20 years). This mummy was then utilized as a representative sample for morphological and analytical investigations.

While macroscopic investigation is significant in the study of mummies, the analysis of mummified tissue is also considered very useful for understanding characteristics of the mummification process. In the literature, morphological analyses are usually found to be performed through conventional scanning electron microscopy (SEM), which has some limits; for example, the samples must be dehydrated and covered with a conductive film before the observation [21,22]. As described by Burattini et al. (2016) [21], after alcohol dehydration, the samples are critical-point dried and gold sputtered; thus, the specimen is subjected to an irreversible treatment and cannot be used for further analysis. Thanks to ESEM, a microscope which works at low vacuum, the sample is not subject to physical disturbance and, therefore, to any artifacts. Mummified tissues are directly deposited on conductive stubs and observed at ESEM.

**Figure 7:**
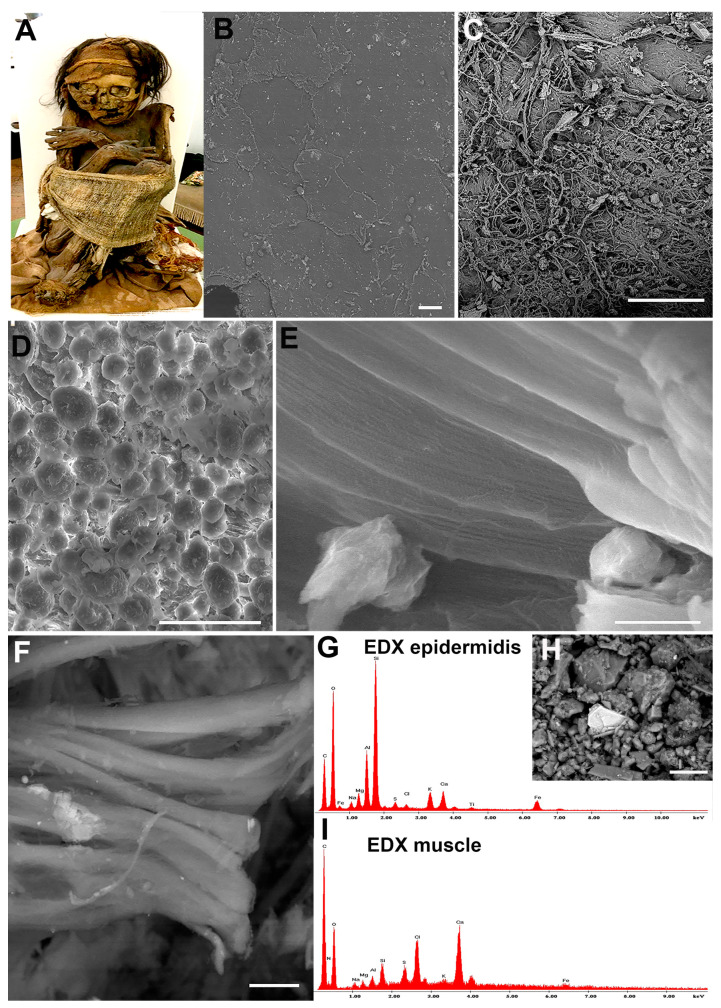
ESEM observation of Peruvian mummy remains (**A**), epidermis (**B**), dermis (**C**), subcutaneous layer (**D**), tendon (**E**), and muscle fibers (**F**). Finally, two representative EDX spectra (**G**,**I**) of adjacent dermal tissue (**H**) reveal the presence of chemical elements, which detail the mummification habitat related to soil and brick. Scale bars: 250 nm for (**B**,**C**); 50 µm for (**D**); 1 µm for (**E**); 10 µm for (**F**); 20 µm for (**H**).

In particular, the state of the preservation of ancient mummified remains from the Peruvian mummy (Figure 7A) was evaluated with ESEM.

Ultrastructural observations revealed a good preservation of mummy tissues. The epidermis showed the presence of keratinocytes (Figure 7B) and the intertwined connective fibers appeared in the dermal layer (Figure 7C). In the subcutaneous layer, we observed the presence of well-preserved fat cells, which appeared round with a size of about 20 μm, comparable to human adipocytes (Figure 7D). Furthermore, preserved tendon (Figure 7E) and muscle fibers (Figure 7F) appeared. Analyzing collagen fibers, which showed a parallel distribution (probably collagen type I, since it is typically located mainly in skin and tendon, and it is the main part of the organic part of bone), a periodic band can be observed, with a preserved banding pattern of about 70 nm. Since ESEM observation has the potential to provide valuable insights into the state of deteriorating remains without altering the samples, it allows for subsequent applications. EDX analysis was then conducted on the same mummified tissues from this Peruvian mummy (Figure 7E) in order to characterize the organic and inorganic elements present in the mummification habitat/environment. For instance, the EDX spectrum (Figure 7G) obtained from skin tissue analysis revealed the presence of silicates, suggesting that the environment in which the mummy was found had been contaminated by the soil. This is demonstrated in Figure 7H, where microscopic stones contaminate the sample. In Figure 7I, the EDX spectrum obtained from muscle is shown and reveals the presence of calcium carbonate, comparable to brick fragments, likely coming from the crypt in which the bodies were buried. In addition, traces of sulfur were detected and could be attributed to the soil, but it was also contained in the muscles.

## 4. Discussion

The abilities of paleoradiologists were greatly enhanced with the introduction of computed tomography (CT) in the early 1970s [24]. CT imaging is widely used in mummy research to non-invasively assess several parameters, such as mummification technique, age at death, bone and soft tissue preservation, artifacts, sex, diseases, traumas, the cause of death, and medical interventions [25,26,27,28,29,30,31]. CT images can be converted into DICOM files and processed through medical image software. Among these revolutionary technologies is Anatomage Table, which was used in the present work for the first time as a virtual imaging tool for the study of ancient corpses, thanks to its distinct ability to display real segmented human anatomy and physiology in 1:1 life-size. With Anatomage, it is possible to replicate a three-dimensional model of mummified bodies through layers of dresses without putting the ancient artifact at risk, representing an advantageous imaging tool for the description of ancient corpses [12,32,33,34]. Furthermore, it can monitor the length of bone segments, the presence of tissue remains such as ligaments and tendons, the presence of skeletal and dental pathologies, and skeletal deformities, allowing researchers to reconstruct the customs and traditions of an ancient population.

Four mummies were observed in Anatomage Table without physically damaging them. First, by examining the attire and accessories adorned by the mummies, it was possible to conjecture details of the customs and traditions of the epoch. Subsequently, the mummies were stripped to the internal organs with tendons, ligaments, and especially bones perfectly recognizable and discernible in all projections. The analysis of teeth detected the presence of caries that can be associated with the food habits of that time. Moreover, the observation of skeleton degeneration revealed very important clues about daily life. For instance, the mummy known as “the dancer” showed a left asymmetry of the lumbar spine, which could be associated to the habit of performing housework while keeping the child in the left arm. This could be assumed by her young age and the absence of additional pathological indicators on other joints. The lumbar spine degeneration of the mummified man (the third mummy) could be associated with loading work compatible with transhumance and charcoal burner, typical activities of the 16-18th century on the high Modena Apennines [35]. All of this information can be obtained starting from a CT image, converting it to DICOM format, and reconstructing the mummy through Anatomage Table, a new imaging technology for training medical students that could be applied in several fields. Furthermore, the anatomical table enables the observation of mummified bodies in real size, offering the possibility to study soft and hard tissues in all anatomical projections with appropriate filters. Anatomage Table offers an easy and fast interface between the operator and a 3D rendering. It is possible to apply cuts that can be transformed into section planes sliding along the three axes. Numerous filters are available, and there is the possibility to enlarge details well beyond the dimensions of a normal monitor. These unique features make it a first-choice analysis tool compared to normal DICOM file management software loaded on a PC, a notebook, or a tablet. In conclusion, Anatomage Table does not replace CT scan images but can be considered an adjunct tool for researchers with several advantages:

- Working in real size and carrying out morphometric analyses without ruining the ancient body;
- Dissecting the body, which allows the observation of the region of interest in all possible projections without damaging the ancient corps;
- Isolating individual anatomical structures and studying them in detail through various filter applications;
- Appreciating the garments of the mummy and removing the clothes gradually, thus being able to highlight details that may escape CT analysis;
- Training of paleopathologist researchers.

Finally, the study of mummified remains is crucial in this research since it allows us to understand tissue preservation and to correlate it to mummification processes. The Peruvian mummy tissues observed at ESEM equipped with EDX analyses revealed the maintained epidermis and dermis by distinguishing the keratinocytes drawn to one another and detecting the dense collagen fibers. It was possible to describe well-preserved adipocytes in the subcutaneous layers, muscle fibers, and tendon structures. These results crucially contribute to the understanding of the mummification preservation process, whereas EDX chemical analysis detects the presence of potential inorganic/organic compounds, and it is useful to understand lifestyle habits and diseases in the past. This analytical approach allows us to understand if a mummy is affected by soil/environmental contamination, yielding more reliable results.

## 5. Conclusions

In conclusion, the study of ancient corpses requires several interdisciplinary approaches, from CT images to virtual dissection and from microscopy to spectroscopy analyses. All these complementary techniques can help the paleopathologist to better explore and describe human mummified bodies.

Furthermore, in recent years, the international scientific community has increasingly recognized the need to study ancient human remains ethically by ensuring their conservation and avoiding non-essential invasive and destructive analyses.

For this reason, it is becoming more important to establish new diagnostic standards for the non-invasive examination of ancient human remains. The combined use of Anatomage Table and ESEM–EDX technology offers valuable insights into the conservation status of the tissues being studied. Additionally, it allows for the virtual manipulation of these remains within a high-performance environment, ensuring an ethical approach to preserving these precious memories of the past.

## Data Availability

Data are contained within the article.
